# Computational analysis to repurpose drugs for COVID-19 based on transcriptional response of host cells to SARS-CoV-2

**DOI:** 10.1186/s12911-020-01373-x

**Published:** 2021-01-07

**Authors:** Fuhai Li, Andrew P. Michelson, Randi Foraker, Ming Zhan, Philip R. O. Payne

**Affiliations:** 1 Institute for Informatics (I2), Washington University in St. Louis School of Medicine, St. Louis, MO USA; 2 Department of Pediatrics, Washington University in St. Louis School of Medicine, St. Louis, MO USA; 3 Pulmonary and Critical Care Medicine, Washington University in St. Louis School of Medicine, St. Louis, MO USA; 4 National Institute of Mental Health (NIMH), NIH, Bethesda, MD USA

## Abstract

**Background:**

The Coronavirus Disease 2019 (COVID-19) pandemic has infected over 10 million people globally with a relatively high mortality rate. There are many therapeutics undergoing clinical trials, but there is no effective vaccine or therapy for treatment thus far. After affected by the Severe Acute Respiratory Syndrome Coronavirus 2 (SARS-CoV-2), molecular signaling pathways of host cells play critical roles during the life cycle of SARS-CoV-2. Thus, it is significant to identify the involved molecular signaling pathways within the host cells. Drugs targeting these molecular signaling pathways could be potentially effective for COVID-19 treatment.

**Methods:**

In this study, we developed a novel integrative analysis approach to identify the related molecular signaling pathways within host cells, and repurposed drugs as potentially effective treatments for COVID-19, based on the transcriptional response of host cells.

**Results:**

We identified activated signaling pathways associated with the infection caused SARS-CoV-2 in human lung epithelial cells through integrative analysis. Then, the activated gene ontologies (GOs) and super GOs were identified. Signaling pathways and GOs such as MAPK, JNK, STAT, ERK, JAK-STAT, IRF7-NFkB signaling, and MYD88/CXCR6 immune signaling were particularly activated. Based on the identified signaling pathways and GOs, a set of potentially effective drugs were repurposed by integrating the drug-target and reverse gene expression data resources. In addition to many drugs being evaluated in clinical trials, the *dexamethasone* was top-ranked in the prediction, which was the first reported drug to be able to significantly reduce the death rate of COVID-19 patients receiving respiratory support.

**Conclusions:**

The integrative genomics data analysis and results can be helpful to understand the associated molecular signaling pathways within host cells, and facilitate the discovery of effective drugs for COVID-19 treatment.

## Background

By June 29, 2020, there were over 2,500,000 confirmed cases (with > 120,000 deaths) of *Coronavirus Disease 2019 (*COVID-19) in the U.S. and over 10 million cases (with > 500,000 deaths) globally, based on the COVID-19 Dashboard [1] operated by the Center for Systems Science and Engineering at Johns Hopkins University (JHU) (https://coronavirus.jhu.edu/map.html). The primary organ of infection is considered to be the lung, and the infection leads to acute hypoxemic respiration and ultimately to multi-organ failure and death [2]. The mortality rate of COVID-19 is relatively high [3], compared with the flu epidemic. So far, there is no newly FDA approved drug for the treatment of COVID-19. Recently, *remdesivir,* developed by Gilead Sciences, was recently approved for COVID-19 treatment. However, *remdesivir* can reduce the time of recovery and cannot significantly reduce the mortality rate [4]. To improve the outcome of COVID-19 patients, many existing drugs are being evaluated in clinical trials globally, like *chloroquine and hydroxychloroquine, azithromycin, and lopinavir–ritonavir, and* dexamethasone*.* Thus, repurposing existing medications is considered an important approach to speed up the drug discovery for COVID-19. A few days ago, dexamethasone, an existing FDA approved drug, was reported to be the first drug that can reduce the death rate, by one-fifth to one-third, of COVID-19 patients receiving respiratory support [5], which was ranked No.1 in our analysis.

Although 1,570 clinical trials have been initiated globally for COVID-19 treatment by June 29, 2020, based on the data from the dashboard [6] of real-time clinical trials of COVID-19 (https://www.covid-trials.org/), only one drug, dexamethasone, was reported to be able to significantly reduce the death rate of COVID-19 patients receiving respiratory support [5]. One possible reason is that most of the current clinical trials are based on limited knowledge of the disease and observed phenotypes. The molecular mechanisms and signaling pathways within the host cells such as lung cells, which play critical roles in the life cycle of *Severe Acute Respiratory Syndrome Coronavirus 2 (*SARS-CoV-2) infection, remain unidentified. Thus, it is significant to uncover the mysterious molecular signaling pathways within host cells via computational data analysis. It is also important and needed to facilitate drug repurposing and design of new clinical trials. For example, in the recent studies [7, 8] the cogena model (co-expressed gene-set enrichment analysis) was applied to identify the co-expressed differentially expressed genes (DEGs) in the gene expression data of Bronchoalveolar Lavage Fluid (BALF) [9] samples of COVID-19 patients, and the activated signaling gene sets in KEGG [10–12] and Reactome [13]. Then two potentially effective anti-viral drugs, saquinavir and ribavirin, were identified using connectivity map (CMAP) database.

In order to better understand the transcriptional response of lung cells to the SARS-CoV-2 infection, Albrecht and tenOever laboratories profiled the RNA-seq gene expression from human NHBE (Normal Human Bronchial Epithelial) cells, A549 lung cancer cells (no ACE2 expression), A549_ACE2 (A549 lung cancer cells transduced with a vector expressing human ACE2), and CALU-3 lung cancer cells (with ACE2 expression), and 2 human lung samples infected by SARS-CoV-2 [14]. The data are valuable sources for identifying genetic pathways and biological processes that become dysregulated during active infection. More importantly, the data allows for the identification of activated signaling pathways that can be targeted by existing pharmaceutical agents. In this study, we aimed to identify activated signaling pathways within lung host cells affected by SARS-CoV-2 and repurpose existing drugs for COVID-19 treatment using a novel integrative data analysis approach, integrating transcriptional response [14], signaling pathway [10], 15, gene ontology [16], drug-target interactions from drugbank [17] and reverse gene signature data from connectivity map (CMAP) [18–20]. These results, including the identified signaling pathways, activated GOs, and drugs, can be helpful to facilitate the experimental screening and clinical trial design to speed up the therapeutic discovery for COVID-19.

## Methods

RNA-seq data (gene expression) from NHBE (Normal Human Bronchial Epithelial) cells, A549 (no ACE2 expression) cells, A549_ACE2 cells (A549 lung cancer cells transduced with a vector expressing human ACE2), and CALU-3 lung cancer cells (with ACE2 expression) cells infected by SARS-CoV-2 were obtained from GEO (GSE147507) [14]. This data was generated by Drs. Albrecht and tenOever’s at the Icahn School of Medicine at Mount Sinai. Specifically, for each cell line, 3 control (no SARS-CoV-2 infected) and 3 SARS-CoV-2 infected samples were used respectively. The DEseq2 [21] tool was used to calculate the fold change and p-value of individual genes in the NHBE (normal tissue), A549_ACE2, and CALU-3 lung cancer cells respectively before- and after- viral exposure. The data of A549 cell was not used considering that the ACE2, with which SARS-CoV-2 interacts to enter host cells, is not expressed in A549 cell.

For the signaling network analysis, the 307 KEGG (Kyoto Encyclopedia of Genes and Genomes) [10–12] signaling pathways were extracted using the ‘graphite’ R package [22, 23]. To identify the activated signaling network for NHBE, A549_ACE2 and CALU-3 cells respectively, the signaling paths, i.e., the shortest paths link source signaling genes (starting genes on the signaling pathways) and the sink signaling genes (ending genes on the signaling pathways) within the 307 KEGG signaling pathways were first identified. For each signaling path, the average fold change of genes on the signaling path was calculated. Then the signaling paths with the average fold change score greater than the mean score + 1.25 standard deviation threshold were selected to construct the activated signaling network. To remove the potential tumor-specific signaling pathways, the common and intersection signaling pathways between NHBE and A549_ACE2 cell lines, and the common signaling between NHBE and CALU-3 cell lines were then combined as the potential activated signaling pathways associated with the viral infection. To identify potential drugs that can inhibit the signaling genes on the activated signaling pathways, the drug-target interactions of FDA-approved drugs were downloaded from the DrugBank [17] database. Then drugs targeting the activated signaling pathways were identified as potential effective drug candidates for COVID-19 treatment.

For the gene ontology (GO) [16] analysis, the Fisher’s exact test, with a threshold p-value = 0.05, was used to identify the statistically activated GOs based on the up-regulated genes. Since there are many activated GOs, and some of them are semantically close and sharing the common set of genes, it was difficult to identify the most important GOs. To solve this challenge, we first manually removed many of the activated GOs that were not related to biological signaling processes or general diseases. In addition, we defined the super-GOs, which are defined as sub-groups of GOs that have similar or related biological processes. Specifically, after the calculation of GO-GO similarity using the semantic similarity [24] (GOSemSim R package) between activated GOs, the affinity propagation clustering [25] (APclustering) was used to divide the activated GOs into sub-groups (named super-GOs). Then, the genes that were up-regulated within each super-GO were used as signatures to identify potential drugs that can inhibit the activation of the super-GOs. These gene signatures were fed into the updated connectivity map (CMAP) [18, 19] database to identify potential drugs, which included the gene expression profiles on a set of cancer cell lines before and after perturbation of 2,513 drugs and compounds. Then the gene set enrichment analysis (GSEA) [26] was applied on the z-profiles (gene expression variation before and after treatment with 2,513 drugs and investigational agents) of 9 cells [18, 19] in the updated CMAP to identify gene set signature-specific inhibitory drugs. The top ranked FDA drugs, based on the average GSEA scores, that can potentially inhibit the up-regulated gene signatures associated with the super-GOs were identified as potential candidates for repurposing for COVID-19 treatment.

## Results

### Activated signaling pathways and associated inhibitory medications

**Fig. 1:**
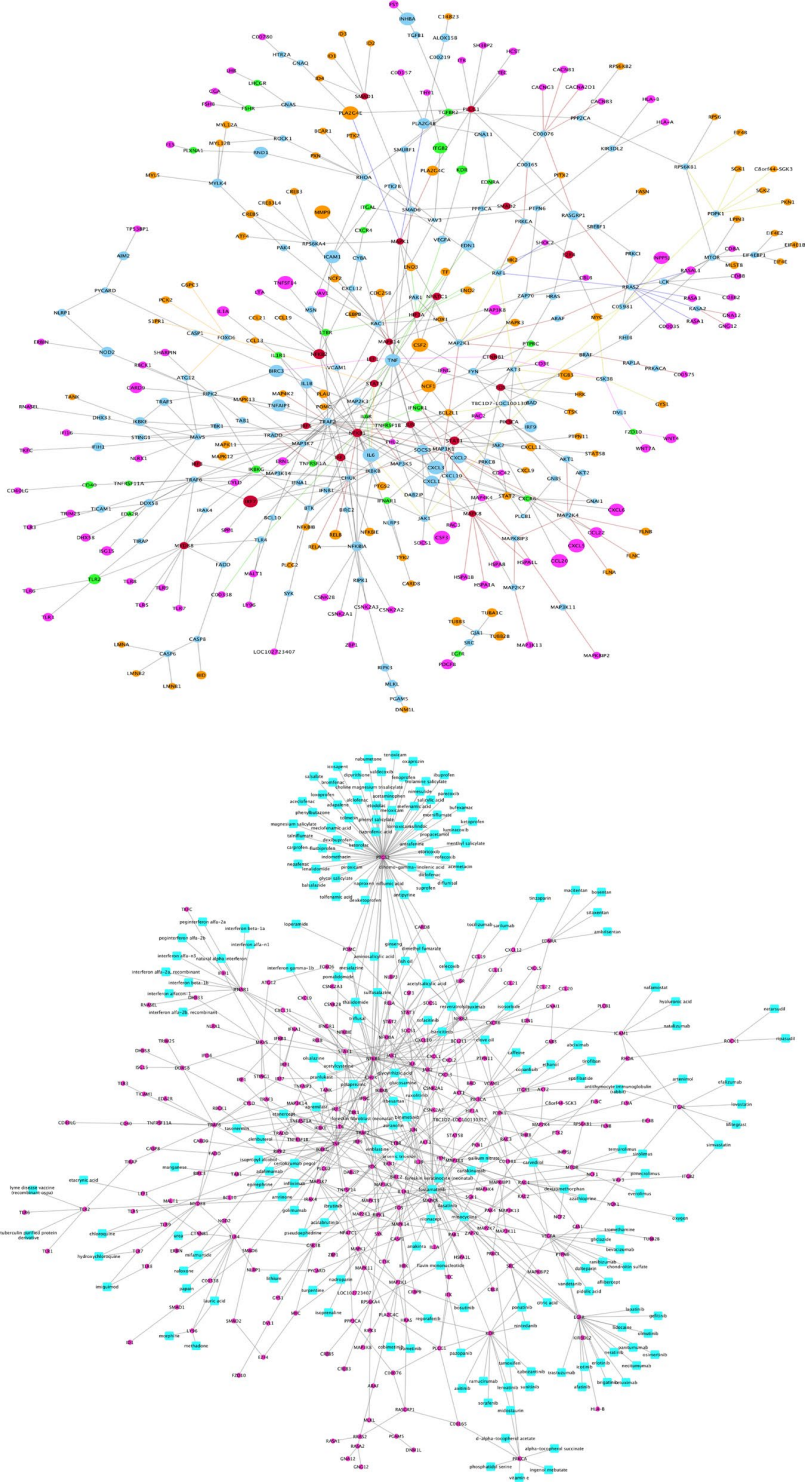
(Upper) Activated signaling pathways (with 244 genes) within NHBE and also appeared in lung A549_ACE2 and CALU3 cells after SARS-CoV-2 infection. Red nodes are transcription factors (TFs); green—receptors; purple—ligands; orange—activated target genes of TFs; and cyan—the linking genes. (Lower) 220 drugs (cyan) (with 97 target genes) targeting on the activated signaling pathways

The KEGG signaling pathway analysis was conducted to identify the potentially activated signaling pathways within the lung host cells after SARS-CoV-2 infection (see Fig. 1-Upper). As seen, the IRF7/IFR9, NFkB1, NFkB2, STAT1, TNF, MAPK3K8, MAPK8, and MAPK14 related signaling pathways that were identified as the major activated transcription factors (TFs), which can potentially activate the activation of other predicted signaling pathways, including those mediated by CXCR6/CXCL1/CXCL2/CXCL3/CXCL10, MYD88, CREBBP, JAK1/JAK2, STAT and MAPK signaling pathways. Also, the WNT4/WNT7A and SMAD signaling pathways are also identified to be activated. Moreover, the PDGFB-EGFR and TUBB1C/2B/3 proliferation signaling pathways are also predicted as the COVID-19 related signaling pathways.

Based on the drug-target interaction data derived from DrugBank and the identified signaling network, 220 drugs were identified to inhibit 97 target genes on the signaling network (with 71 drugs targeting the PTGS2 gene specifically) (see Additional file 1: Table S1). As seen, Chloroquine and hydroxychloroquine were found to be able to potentially inhibit MYD88 immune signaling based on their targets, TLR7 and TLR9 that directly interact with the MYD88 target on the signaling network. Also, acetylsalicylic acid, thalidomide, pranlukast, triflusal, glycyrrhizic acid and fish oil have targets in NFkB signaling pathway. Some reported studies showed the importance of predicted signaling pathways. For example, the NFkB signaling pathway was previously reported as a potential signaling pathway target for SARS [27] treatment, and can also potentially inhibit IRF7 activity. For example, the drug thalidomide, inhibiting NFkB and TNF, was reported as potential treatment for COVID-19 [28]. Moreover, the tumor necrosis factor (TNF) identified in this study was reported in the Lancet [29] to be an important therapeutic target for COVID-19. Also, JAK1/2 pathways were also reported as important targets, and their inhibitors, ruxolitinib, tofacitinib, baricitinib and fostamatinib, could be effective for COVID-19 treatment. Particularly, baricitinib, an arthritis drug, could help reduce the out-of-control immune response (https://www.wired.com/story/ai-uncovers-potential-treatment-covid-19-patients/, and https://www.clinicaltrialsarena.com/news/eli-lilly-to-study-baricitinib-for-covid-19-treatment/), was reported in the Lancet [30] as a COVID-19 suitable treatment. In addition, the MAPK1, AKT and PRKCA inhibitors such as isoprenaline, arsenic trioxide, vitamin e, and midostaurin could be also effective. Moreover, the IL6R inhibitor tocilizumab was reported for COVID-19 treatment [31], and another IL6R inhibitor, sarilumab, is being evaluated in clinical trials (ClinicalTrials.gov Identifier: NCT04327388). Lastly, STAT1 and IFANR1 were identified as potential targets for COVID-19 treatment and were reported by another group as well [32]. These evidences support the predicted signaling pathways. In summary, the identified activated signaling pathways provided potential molecular mechanisms that facilitate the viral replication life cycle, and thus can be potential therapeutic targets to identify effective drugs for COVID-19.

Moreover, the identified drugs inhibiting the predicted signaling pathways were compared with drugs used in the clinical trials for COVID-19 treatment. Specifically, drugs that have been tested or are currently being tested in clinical trials globally were identified from the covid-trials dashboard (Data: table_trials-2020-05-27 05_38_12.csv, and the updated drugs: table_trials-2020-06-29 22_20_46.csv. Some drugs cannot be found in the table_trials-2020-06-29 22_20_46.csv because drug names were replaced by using category names, e.g., *ruxolitinib was replaced by* JAK inhibitor), and compared with the drugs identified from the signaling pathway analysis. Based on the dashboard of clinical trials for COVID-19 treatment, 114 FDA approved drugs were reported from 1,132 clinical trials globally (see Table 1). Among the 108 drugs, 31 of them are in the predicted drugs list. These drugs are: *hydroxychloroquine, chloroquine, tocilizumab, sarilumab, canakinumab, ruxolitinib, oxygen, sirolimus, ibuprofen, anakinra, acalabrutinib, baricitinib, ibrutinib, lenalidomide, tirofiban, acetylsalicylicacid, simvastatin, siltuximab, bevacizumab, tinzaparin, naproxen, celecoxib, tofacitinib, adalimumab, thalidomide, dalteparin, nadroparin, minocycline, lithium, indomethacin, and nintedanib*. In summary, the predicted signaling network could be helpful to understand the molecular mechanisms within lung host cells after SARS-CoV-2 infection. Drugs targets on the signaling targets might be effective (some might also have harmful effects and cautions). Drug combinations (drug cocktails) targeting on different targets can be potentially synergistic for COVID-19 treatment.

**Table 1:** FDA approved drugs in clinical trials for COVID-19 treatment

Zidovudine	Linagliptin	Chlorpromazine	Bevacizumab	Sofosbuvir
Hydroxychloroquine	Telmisartan	Lenalidomide	Tinzaparin	Vitamina
Lopinavir	Anakinra	Methotrexate	Naproxen	Metformin
Tocilizumab	Vitaminc	Tirofiban	Ritonavir	Berberine
Sarilumab	Zinc	Clopidogrel	Tacrolimus	Licorice
Atazanavir	Almitrine	Acetylsalicylicacid	Celecoxib	Bromhexine
Tranexamicacid	Sitagliptin	Fondaparinux	Tofacitinib	Minocycline
Alteplase	Ciclesonide	Ramipril	Pirfenidone	Lithium
Canakinumab	Acalabrutinib	Progesterone	Hydrogenperoxide	Formoterol
Ruxolitinib	Etoposide	Captopril	Sildenafil	Indomethacin
Colchicine	Ketamine	Eculizumab	Ixekizumab	Selenium
Leflunomide	Losartan	Sevoflurane	Dexmedetomidine	Nintedanib
Oxygen	Valsartan	Nitazoxanide	Lipoicacid	Spironolactone
Sirolimus	Baricitinib	Sargramostim	Tranilast	Imatinib
Povidone-iodine	Fluoxetine	Ribavirin	Adalimumab	Estradiol
Fluvoxamine	Vitamind	Nivolumab	Thalidomide	Chloroquine
Ibuprofen	Bicalutamide	Melatonin	Fingolimod	Azithromycin
Aviptadil	Ivermectin	Simvastatin	Suramin	Dexamethasone
Doxycycline	Sodiumbicarbonate	Dapagliflozin	Itraconazole	Oseltamivir
Enoxaparin	Ibrutinib	Amiodarone	Mefloquine	Amoxicillin
Prazosin	Levamisole	Verapamil	Dalteparin	Clavulanate
Isotretinoin	Deferoxamine	Siltuximab	Nadroparin	Darunavir
Heparin	Methyleneblue	Defibrotide	Iloprost	

### Activated gene ontologies (GOs)

Based on the fold change and p-value obtained from the DEseq2 analysis of NHBE, A549_ACE2, and CALU-3 cells before and after the viral infection, the up-regulated genes in each cell were identified respectively. Since only a small number of up-regulated genes will be obtained for NHBE cells using threshold of fold change 2.0. We set the fold-change threshold as 1.25 for the NHBE cell line empirically. Specifically, for the NHBE cells, 558 genes had a statistically significant increase with a fold-change >  = 1.25 with a p value <  = 0.05. For the A549_ACE2 cells, 916 up-regulated genes were identified with a fold-change >  = 2.0 and with a p value <  = 0.05. For the CALU-3 cells, 1335 up-regulated genes were identified with a fold-change >  = 2.0 and with a p value <  = 0.05.

**Fig. 2:**
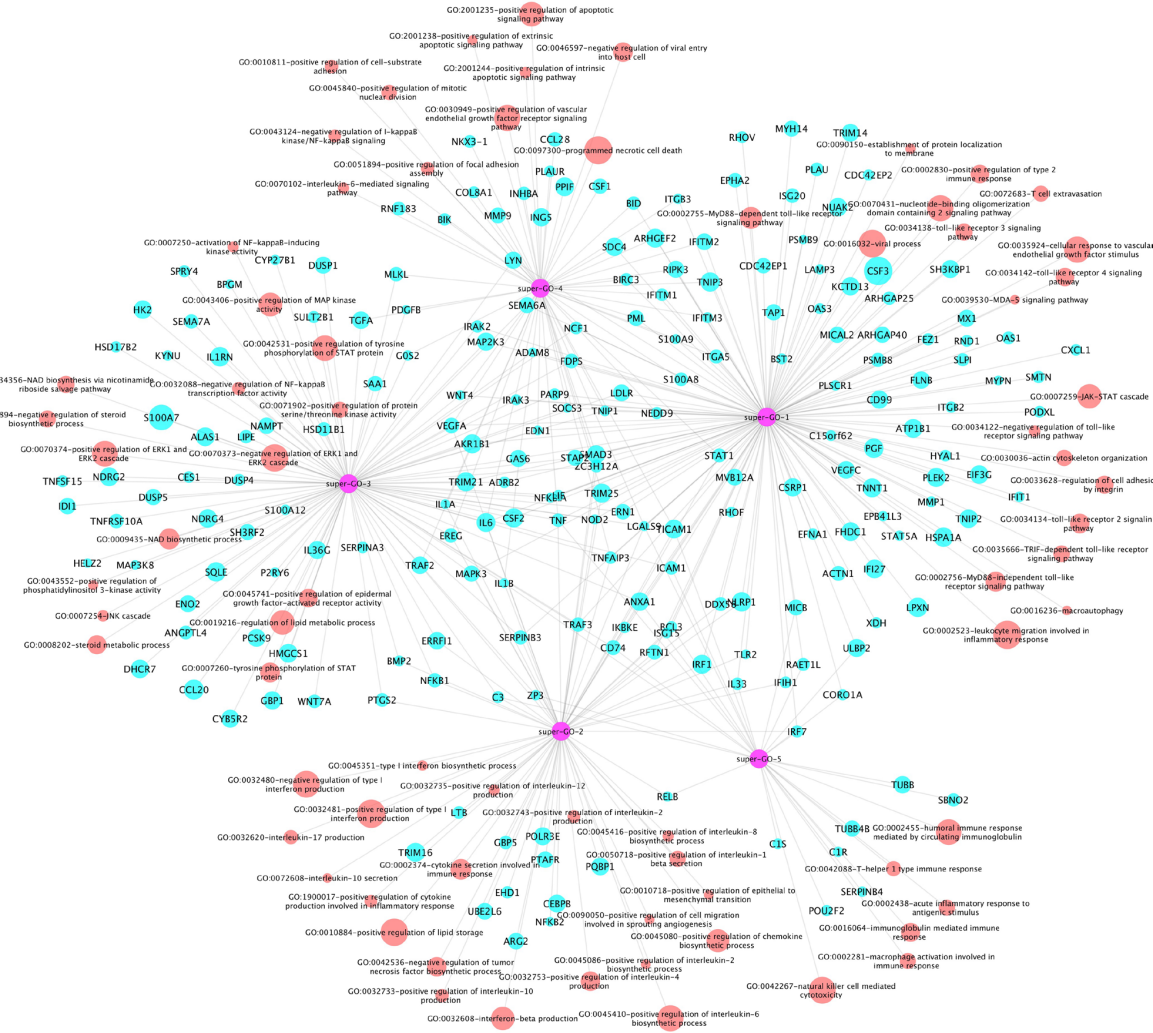
Network of 212 up-regulated genes (cyan color) in lung NHBE, A549_ACE2 and CALU-3 cells after SARS-CoV-2. There are 73 activated gene ontologies (GOs; red color), and 5 super-GOs (clusters of GOs; purple color). The node size of genes and GOs is proportional to the fold change and negative log2 p-values respectively

Based on the three up-regulated gene sets, the activated GOs with enriched genes in the three gene sets were identified respectively with a p-value <  = 0.05 (obtained from the Fisher’s exact test) and the number of genes in GOs between 10 and 1000. Then the common activated GOs between NHBE and A549_ACE2 cells and between NHBE and CALU-3 cells were unified. After empirically removing the unrelated and general GOs, 73 GOs were kept. The full list of enriched GOs was provided in the supplementary file (Cell-line-GO.txt). Then, the clustering analysis was employed to divide the activated GOs into 5 sub-groups (named super-GOs). Among the 73 GOs, 212 up-regulated genes were kept. Figure 2 shows the network of the 212 up-regulated genes, 73 activated GOs and 5 super-GOs. Genes associated with the GOs in each super-GO are collected as gene set signatures for drug discovery analysis. In addition to the viral process related signaling, the GO analysis identified many potential viral infections related signaling pathways, e.g., MAPK, JNK, STAT, ERK1/2, MYD88 and Toll like receptor signaling pathways. These results are consistent and complement the KEGG signaling pathway analysis.

### Repurposing drugs inhibiting individual super-GOs

**Fig. 3:**
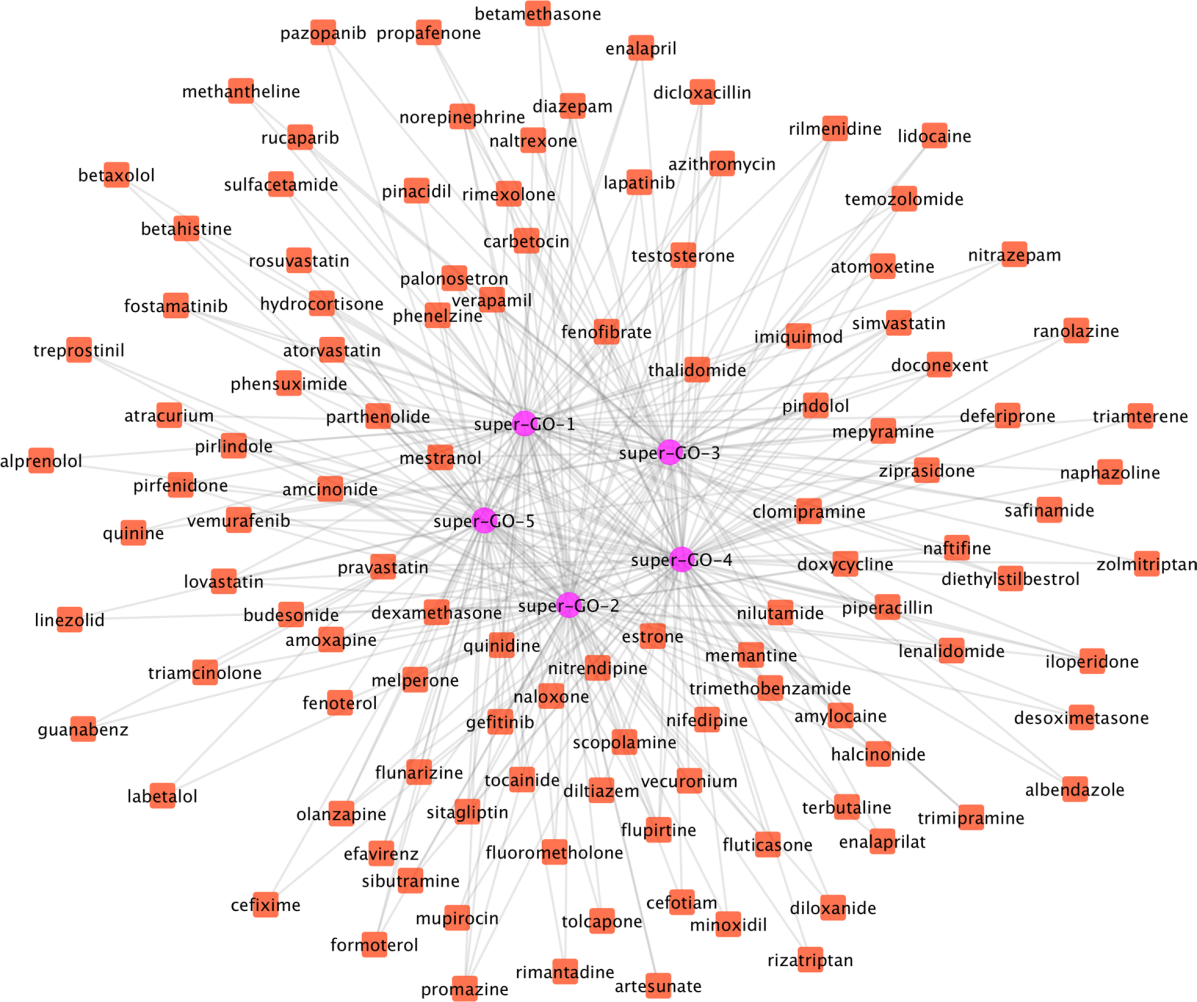
The 113 drugs inhibiting the up-regulated genes in 5 super-GOs

**Fig. 4:**
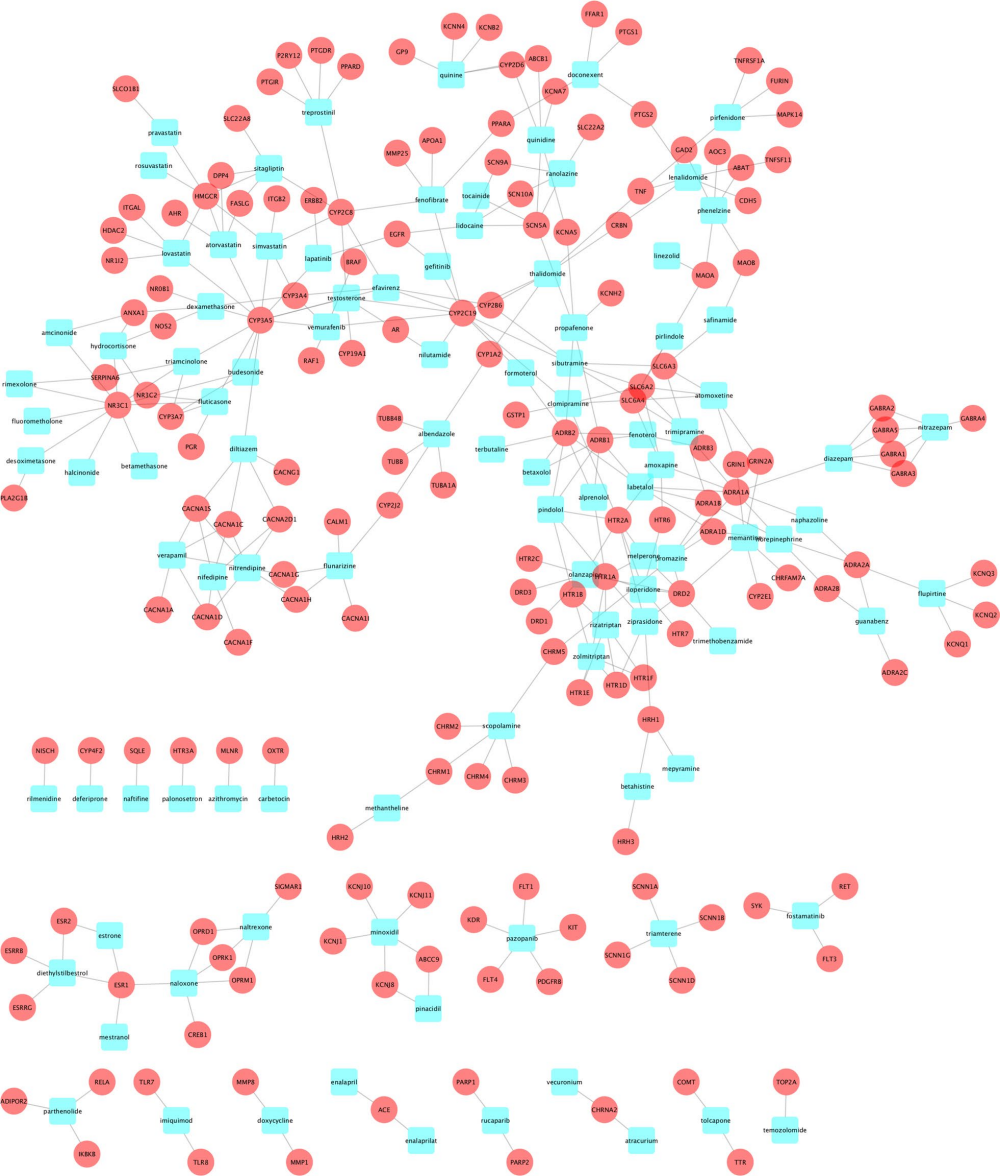
The targets of the 102 (out of 113) drugs inhibiting the up-regulated genes in 5 super-GOs

The gene signatures in each of 5 super-GO clusters were used as the query input of the CMAP database to identify drugs capable of inhibiting the associated genetic pathways. Specifically, 258 drugs appeared in the top 100 drugs in at least one of the 5 super-GOs. There were 26 common drugs among the total drugs derived from aforementioned signaling network analysis and the super-GO analysis (see Table 2). Then 113 drugs were selected based on their frequency (frequency >  = 2) appeared in the top 100 drugs of each of the 5 super-GOs (see Fig. 3 and Table 3). Surprisingly, the dexamethasone (glucocorticoid receptor agonist, corticosteroid agonist, immunosuppressant) (frequency = 5, top ranked) in our prediction, see Table 3), an existing FDA approved drug, was recently reported to be the first drug that can significantly reduce the death rate of COVID-19 patients receiving respiratory support [5]. Specifically, the clinical trials results indicated that dexamethasone reduced death rate by one-third in patients receiving invasive mechanical ventilation, and reduced the death rate by one-fifth in COVID-19 patients receiving oxygen without invasive mechanical ventilation [5]. Moreover, a few top-ranked drugs were reported to be potentially able to treat or reduce the mortality of COVID-19. The fenofibrate, was used in clinical trials for COVID-19 as a metabolic intervention (ClinicalTrials.gov Identifier: NCT04517396). The parthenolide was reported as a potential inhibitor for the cytokine storm [33]. The diabetes drug, sitagliptin, was reported to be associated with reduced mortality in COVID-19 patients with type 2 diabetes. Also the stains drugs, like atorvastatin and lovastatin, was associated with reduced hazard for fatal or severe disease of COVID-19. Also, hydrocortisone (corticosteroid agonist, glucocorticoid receptor agonist, immunosuppressant, interleukin receptor antagonist) (frequency = 3) were reported to be related to COVID-19 treatment [34]. To help understand the potential mechanisms of the drugs, the 102 drugs (some drugs have no target information) and 170 targets interaction network was provided in Fig. 4. We further compared the GO analysis derived drugs with the clinical trials drugs, and 10 overlapping drugs were in both the prediction list and the clinical trial reports. The 10 drugs are: dexamethasone, sitagliptin, azithromycin, doxycycline, simvastatin, verapamil, formoterol, lenalidomide, pirfenidone, thalidomide. Moreover, 3 drugs appeared in the predictions derived from signaling network analysis, GO analysis and clinical trials: lenalidomide, simvastatin, thalidomide. In summary, the evidence of reported studies and clinical trials indicated that the prediction analysis could be potentially helpful for repurposing existing drugs as potentially effective treatments for COVID-19.

**Table 2:** Twenty-seven common FDA approved drugs derived from signaling network analysis and super-GO analysis

Caffeine	Lenalidomide	Naproxen	Sunitinib	Talniflumate
Lovastatin	Dextromethorphan	Phenylbutazone	Resveratrol	Fostamatinib
Lidocaine	Diclofenac	Thalidomide	Nimesulide	Chloroquine
Gefitinib	Chloroquine	Naloxone	Pazopanib	
Sorafenib	Simvastatin	Dasatinib	Tofacitinib	
Nabumetone	Imiquimod	Lapatinib	Afatinib

**Table 3:** Top 113 drugs frequently appeared in the top-ranked drugs of 5 super-GOs

Name	Freq	Name	Freq	Name	Freq	Name	Freq
Dexamethasone	5	Amcinonide	3	Quinidine	3	Minoxidil	2
Atorvastatin	5	Amoxapine	3	Rosuvastatin	3	Mupirocin	2
Fenofibrate	5	Amylocaine	3	Scopolamine	3	Naphazoline	2
Flupirtine	5	Budesonide	3	Temozolomide	3	Nilutamide	2
Palonosetron	5	Dicloxacillin	3	Thalidomide	3	Nitrazepam	2
Parthenolide	5	Diethylstilbestrol	3	Tocainide	3	Pazopanib	2
Pindolol	5	Diltiazem	3	Albendazole	2	Phenelzine	2
Sitagliptin	5	Doconexent	3	Alprenolol	2	Pinacidil	2
Trimethobenzamide	5	Efavirenz	3	Artesunate	2	Propafenone	2
Vemurafenib	5	Enalapril	3	Atomoxetine	2	Quinine	2
Azithromycin	4	Estrone	3	Atracurium	2	Ranolazine	2
Carbetocin	4	Fluticasone	3	Betahistine	2	Rimantadine	2
Deferiprone	4	Formoterol	3	Betamethasone	2	Rimexolone	2
Diazepam	4	Fostamatinib	3	Betaxolol	2	Rizatriptan	2
Doxycycline	4	Hydrocortisone	3	Cefixime	2	Rucaparib	2
Gefitinib	4	Lapatinib	3	Cefotiam	2	Safinamide	2
Halcinonide	4	Lenalidomide	3	Clomipramine	2	Sibutramine	2
Iloperidone	4	Mepyramine	3	Desoximetasone	2	Sulfacetamide	2
Lovastatin	4	Naftifine	3	Diloxanide	2	Terbutaline	2
Melperone	4	Naloxone	3	Enalaprilat	2	Tolcapone	2
Memantine	4	Naltrexone	3	Fenoterol	2	Treprostinil	2
Mestranol	4	Nifedipine	3	Flunarizine	2	Triamcinolone	2
Norepinephrine	4	Nitrendipine	3	Fluorometholone	2	Triamterene	2
Promazine	4	Olanzapine	3	Guanabenz	2	Trimipramine	2
Rilmenidine	4	Phensuximide	3	Imiquimod	2	Vecuronium	2
Simvastatin	4	Piperacillin	3	Labetalol	2	Zolmitriptan	2
Testosterone	4	Pirfenidone	3	Lidocaine	2		
Verapamil	4	Pirlindole	3	Linezolid	2		
Ziprasidone	4	Pravastatin	3	Methantheline	2

### Activated GO terms and associated drugs using human tissue samples of COVID-19

Human lung biopsies in the same GSE147507 dataset and Bronchoalveolar Lavage Fluid (BALF) samples [9] of COVID-19 patients were analyzed to identify activated GO terms and associated drug for potential repurposing. There were 660 up-regulated genes in Human lung biopsies with fold change >  = 2.0 and p-value <  = 0.05 obtained by using DESeq2 (2 normal control and 2 covid-19 human samples). In the BALF samples, 1014 up-regulated genes had been identified in the study [9]. By applying the GO enrichment analysis, 86 overlapping GOs were identified; and 51 GOs were manually selected (see Table 4). The full list of enriched GOs was provided in the supplementary file (Human-sample-GO.txt). A seen, many general viral response, cytokines and immune response GO terms, like type I interferon, interleukin-1 and toll-like receptor signaling, were strongly activated.

**Table 4:** Manually selected 51 activated GO terms using human tissue samples

go_id	go_term_name	p.value			
GO:0016032	Viral process	7.75E−08	GO:0002221	Pattern recognition receptor signaling pathway	6.55E−15
GO:0034340	Response to type I interferon	1.78E−15	GO:0034341	Response to interferon-gamma	8.43E−25
GO:0042742	Defense response to bacterium	4.74E −13	GO:0035455	Response to interferon-alpha	8.74E −07
GO:0045071	Negative regulation of viral genome replication	2.38E −14	GO:0035456	Response to interferon-beta	9.80E−06
GO:0051607	Defense response to virus	1.54E−24	GO:0046597	Negative regulation of viral entry into host cell	0.00040034
GO:0060337	Type I interferon signaling pathway	6.45E−16	GO:0039530	MDA-5 signaling pathway	0.00446572
GO:0009615	Response to virus	1.60E−28	GO:0060326	Cell chemotaxis	4.46E−19
GO:0045088	Regulation of innate immune response	1.92E−18	GO:0048247	Lymphocyte chemotaxis	1.49E−11
GO:0098586	Cellular response to virus	0.00074571	GO:0006959	Humoral immune response	2.95E−16
GO:0071347	Cellular response to interleukin-1	7.13E−07	GO:0006935	Chemotaxis	1.01E−10
GO:0071356	Cellular response to tumor necrosis factor	4.84E−07	GO:0019731	Antibacterial humoral response	0.00133157
GO:1990869	Cellular response to chemokine	3.29E−08	GO:0002237	Response to molecule of bacterial origin	4.05E−16
GO:0043123	Positive regulation of I-kappaB kinase/NF-kappaB signaling	1.01E−06	GO:0001817	Regulation of cytokine production	3.41E−25
GO:0009617	Response to bacterium	2.67E−25	GO:0051591	Response to cAMP	0.00254496
GO:0071346	Cellular response to interferon-gamma	4.49E−22	GO:0019079	Viral genome replication	4.70E−10
GO:0045089	Positive regulation of innate immune response	1.27E−15	GO:0031663	Lipopolysaccharid E-mediated signaling pathway	1.10E−08
GO:0002224	Toll-like receptor signaling pathway	1.09E−07	GO:0048245	Eosinophil chemotaxis	4.79E−05
GO:0050832	Defense response to fungus	0.00026658	GO:0070098	Chemokin E-mediated signaling pathway	4.75E−08
GO:0002548	Monocyte chemotaxis	6.18E−09	GO:0045055	Regulated exocytosis	1.68E−24
GO:0002218	Activation of innate immune response	9.08E−15	GO:0007249	I-kappaB kinase/NF-kappaB signaling	7.22E−08
GO:0034612	Response to tumor necrosis factor	2.65E−07	GO:0043122	Regulation of I-kappaB kinase/NF-kappaB signaling	1.61E−07
GO:0070555	Response to interleukin-1	5.34E−07	GO:0039528	cytoplasmic pattern Recognition receptor signaling pathway in response to virus	0.01386028
GO:0002430	Complement receptor mediated signaling pathway	2.11E−05	GO:0050920	Regulation of chemotaxis	1.71E−08
GO:0050688	Regulation of defense response to virus	3.49E−06	GO:0030595	Leukocyte chemotaxis	3.11E−20
GO:0071357	Cellular response to type I interferon	6.45E−16	GO:0019058	Viral life cycle	1.15E−09
GO:0001819	Positive regulation of cytokine production	5.35E−15			

To identify drugs using updated CMAP database, the 51 GO terms were clustered into five super-GOs. Then genes associated with individual super-GOs were used as the signatures to identify the associated drugs that can potentially inhibit the individual GOs. In total, 120 drugs were identified that appeared at least two times in the top 100 drugs associated with the five super-GOs. Importantly, there were 49 overlapping drugs between the drug lists identified the super-GO gene signatures of cell lines infected by SARS-CoV-2 and human tissue samples of COVID-19 respectively (see Table 5). These overlapping drugs can be potentially effective to inhibit the cytokine and immune responses to inhibit the cytokine storm of COVID-19.

**Table 5:** 49 common top-ranked drugs identified using the super-GO gene signatures of cell lines infected by SARS-CoV-2 and human tissue samples of COVID-19 respectively

Dexamethasone	Promazine	Lenalidomide	Tocainide	Propafenone
Atorvastatin	Rilmenidine	Mepyramine	Albendazole	Rizatriptan
Palonosetron	Testosterone	Naloxone	Betahistine	Rucaparib
Sitagliptin	Verapamil	Naltrexone	Betamethasone	Safinamide
Trimethobenzamide	Amylocaine	Nifedipine	Betaxolol	Sibutramine
Vemurafenib	Enalapril	Olanzapine	Fenoterol	Terbutaline
Iloperidone	Estrone	Phensuximide	Labetalol	Treprostinil
Melperone	Fluticasone	Pirlindole	Minoxidil	Triamcinolone
Memantine	Hydrocortisone	Scopolamine	Nitrazepam	Vecuronium
Norepinephrine	Lapatinib	Temozolomide	Pinacidil	

### Identify potentially effective drugs using the up- and down-regulated genes

In addition, we also investigated drug prediction using the up- and down-regulated genes directly, which can provide additional evidence to identify potentially effective drugs. For the cell lines, the common up- and down-regulated genes between NHBE and CALU-3 cell lines were used because there were much fewer common genes among all three cell lines (NHBE, A549_ACE2, CALU-3). Also, the top 180 common up- and 183 common down-regulated genes between NHBE and CALU-3 cell lines were selected to identify potentially effective drugs in the CMAP database. Specifically, 30 out of 180 up-regulated genes, and 39 out of 183 down-regulated genes were not available in the CMAP database. Thus 150 up-regulated and 144 down-regulated genes were used. Among the top 100 FDA drugs, there were 13 drugs also appeared in the clinical trials, i.e., sildenafil, lenalidomide, dexamethasone, sitagliptin, simvastatin, azithromycin, formoterol, thalidomide, fluoxetine, lopinavir, valsartan, verapamil, chloroquine. The best rank of dexamethasone was 6. The prediction is similar to the prediction using GO term analysis. Therefore, the prediction can be used as additional evidence of how these drugs can potentially reverse the differentially expressed genes.

For the human samples, there were 156 common up-regulated genes and 162 common down-regulated genes between the human lung tissues and BALF samples. respectively. The common up- and down-regulated genes were used to identify potentially effective drugs in the CMAP database. Specifically, 31 out of 156 up-regulated genes, and 15 out of 162 down-regulated genes were not available in the CMAP database. Thus 125 up-regulated and 147 down-regulated genes were used. Among the top 100 FDA drugs, there were 5 drugs also appeared in the clinical trials, i.e., valsartan, doxycycline, isotretinoin, metformin, progesterone. The best rank of dexamethasone was 330 (not top-ranked). The results are not consistent with the GO-term based analysis. One possible reason is that much noisy genes were identified in the human tissue and BALF samples as signatures to identify the related drugs in the CMAP database. Therefore, GO term analysis is helpful to identify biologically meaningful gene sets as gene set signatures to identify drugs in CMAP database.

## Discussion and conclusions

Currently, there is no effective new drugs or vaccine approved for the treatment of COVID-19, though rapid developments are occurring and being translated into clinical use. As the disease continues to spread, it is becoming increasingly important to develop a treatment modality in the most time-efficient manner. One such approach is to use genetic information to inform the repurposing of available medications. This preliminary and exploratory analysis uses transcriptional response (gene expression) profiles from human host cells before and after the infection with the SARS-CoV-2. In the analysis results, some potentially important targets, signaling pathways, and a set of GOs activated within host cells after viral infection, were identified. Moreover, a set of drugs registered for COVID-19 treatment globally were also identified in the analysis. These discoveries can be helpful to facilitate the design of future clinical trials for the COVID-19 treatment.

This exploratory computational study still has some limitations that can be further improved in the future work. First, the genetic data are derived from an in vitro analysis and will inevitably have a gene expression profile different from an in vivo* epithelial,* which may be further modified on a person-to-person basis. Second, the signaling network analysis is a long-standing challenge, and the models could be improved by integrating the KEGG signaling pathways with the GOs (to include more genes) and also incorporating transcriptomic response data of cells and human tissue samples of COVID-19 patients to uncover the core signaling networks involved in the life cycle of SARS-CoV-2 within host cells. Third, the unbiased list of medications generated and presented was not filtered by route of administration or clinical applicability. For instance, the immune dampening chemotherapeutic agent docetaxel was identified, but would likely not be administered to an infected patient due to concern for augmenting viral replication. Therefore, further pipelines and additional information are needed to analyze the potential effects of these medications, and to continue developing this computational approach to medication repurposing. In addition, we will investigate both up-regulated and down-regulated genes, which could be helpful to understand the potential mechanism of viral-host signaling interactions. Network analysis and its applications for drug and drug combination prediction are challenging problems [35–38]. It can be interesting to conduct significance testing of activated signaling pathways, e.g., possibly using a set of randomly generated signaling paths, to identify most important signaling pathways for drug and drug combination prediction. In the future work, we will investigate these challenges.

## Supplementary information

**Additional file 1**. Table S1: Drugs inhibiting targets on the signaling network.

## Data Availability

Gene expression data of cell lines, and human lung tissue was available at Gene Expression Omnibus (GEO) (with the access number: GSE147507): https://www.ncbi.nlm.nih.gov/geo/query/acc.cgi?acc=GSE147507. Differentially expressed genes of human BALF samples were available at (Table S3): https://www.cell.com/cell-host-microbe/fulltext/S1931-3128(20)30244-4?_returnURL=https%3A%2F%2Flinkinghub.elsevier.com%2Fretrieve%2Fpii%2FS1931312820302444%3Fshowall%3Dtrue#supplementaryMaterial. DrugBank: https://go.drugbank.com/. Connectivity Map database: clue.io. KEGG signaling pathway database: https://www.genome.jp/kegg/pathway.html. KEGG signaling pathways are accessible using ‘graphite’ R package: https://bioconductor.org/packages/release/bioc/html/graphite.html. Gene oncology (GO) terms are accessible using ‘GO.db’ R package: https://bioconductor.org/packages/release/data/annotation/html/GO.db.html

## Abbreviations

COVID-19: Coronavirus disease 2019
SARS-CoV-2: Severe acute respiratory syndrome coronavirus 2
GSEA: Gene set enrichment analysis
CMAP: Connectivity map
KEGG: Kyoto Encyclopedia of genes and genomes
GO: Gene ontology
