# Supplementary material for: Computational analysis to repurpose drugs for COVID-19 based on transcriptional response of host cells to SARS-CoV-2
Source: BMC Med Inform Decis Mak. 2021 Jan 7;21:15. doi: 10.1186/s12911-020-01373-x (PMC7789899; doi:10.1186/s12911-020-01373-x)
Supplement: Supplementary file 1 — Additional file 1. Table S1: Drugs inhibiting targets on the signaling network. [file 12911_2020_1373_MOESM1_ESM.docx]

**Table S1**: 220 drugs and targets on the potential dysfunctional signaling network of COVID19.

| **Gene Symbol** | **Drug Name** | **Gene Symbol** | **Drug Name** | **Gene Symbol** | **Drug Name** | **Gene Symbol** | **Drug Name** |
| --- | --- | --- | --- | --- | --- | --- | --- |
| BCL2L1 | isosorbide | IL1A | rilonacept | NCF1 | dextromethorphan | RELA | dimethyl fumarate |
| CASP1 | minocycline | IRAK4 | fostamatinib | NCF2 | dextromethorphan | RIPK1 | fostamatinib |
| CSNK2A2 | fostamatinib | ITGB2 | simvastatin | NFATC1 | pseudoephedrine | RIPK2 | fostamatinib |
| CTNNB1 | urea | LEF1 | etacrynic acid | NFKBIA | acetylsalicylic acid | RPS6KA4 | flavin mononucleotide |
| CXCL10 | clove oil | LTA | etanercept | NOD2 | mifamurtide | SGK1 | flavin mononucleotide |
| CXCL12 | tinzaparin | MAP2K3 | fostamatinib | NOX1 | oxygen | STAT5B | dasatinib |
| ERN1 | fostamatinib | MAP3K11 | fostamatinib | PAK1 | fostamatinib | SYK | fostamatinib |
| FLNA | artenimol | MAP3K13 | fostamatinib | PAK4 | fostamatinib | TAB1 | manganese |
| GJA1 | carvedilol | MAP4K4 | fostamatinib | PKN1 | fostamatinib | TBK1 | fostamatinib |
| HIF1A | carvedilol | MAPK11 | regorafenib | POMC | loperamide | TEC | fostamatinib |
| IFNGR1 | interferon gamma-1b | MAPK13 | fostamatinib | PTK2 | fostamatinib | TLR8 | imiquimod |
| IKBKE | fostamatinib | MYC | nadroparin | RAC2 | dextromethorphan | TNFRSF1A | tasonermin |
| ZAP70 | fostamatinib | CSNK2A1 | resveratrol, fostamatinib | FYN | dasatinib, fostamatinib | IL1R1 | anakinra, foreskin fibroblast (neonatal) |
| AKT1 | arsenic trioxide, resveratrol | FOS | pseudoephedrine, nadroparin | GSK3B | lithium, fostamatinib | IL6R | tocilizumab, sarilumab |
| ITK | pazopanib, fostamatinib | MAPK14 | dasatinib, fostamatinib | PIK3CA | caffeine, copanlisib | RAC1 | dextromethorphan, azathioprine |
| MAP3K1 | binimetinib, fostamatinib | PDPK1 | celecoxib, fostamatinib | PRKCI | tamoxifen, fostamatinib | ROCK1 | ripasudil, netarsudil |
| TLR2 | lyme disease vaccine (recombinant ospa), tuberculin purified protein derivative | TNFRSF1B | etanercept, tasonermin | MAP2K1 | cobimetinib, bosutinib, trametinib, | BTK | dasatinib, ibrutinib, acalabrutinib, fostamatinib |
| TLR7 | imiquimod, hydroxychloroquine | ICAM1 | natalizumab, hyaluronic acid, nafamostat, | MAPK1 | isoprenaline, arsenic trioxide, turpentine, | CHUK | aminosalicylic acid, mesalazine, sulfasalazine, acetylcysteine |
| TLR9 | chloroquine, hydroxychloroquine | LY96 | morphine, methadone, lauric acid, | VCAM1 | ethanol, carvedilol, clove oil, | JAK1 | ruxolitinib, tofacitinib, baricitinib, fostamatinib |
| JAK2 | ruxolitinib, tofacitinib, baricitinib, fostamatinib | TLR4 | naloxone, lauric acid, papain, mifamurtide | ITGAL | efalizumab, antithymocyte immunoglobulin (rabbit), lovastatin, simvastatin, lifitegrast, | IL1B | minocycline, gallium nitrate, canakinumab, rilonacept, foreskin keratinocyte (neonatal), binimetinib |
| JUN | vinblastine, pseudoephedrine, irbesartan, arsenic trioxide | EDNRA | bosentan, acetylsalicylic acid, sitaxentan, ambrisentan, macitentan, | ITGB3 | abciximab, eptifibatide, antithymocyte immunoglobulin (rabbit), tirofiban, resveratrol, | IL6 | ginseng, siltuximab, polaprezinc, foreskin fibroblast (neonatal), foreskin keratinocyte (neonatal), binimetinib |
| NFKB2 | acetylsalicylic acid, glucosamine, glycyrrhizic acid, fish oil | IFNG | olsalazine, glucosamine, apremilast, foreskin fibroblast (neonatal), foreskin keratinocyte (neonatal), | MTOR | pimecrolimus, sirolimus, everolimus, temsirolimus, fostamatinib, | NFKB1 | acetylsalicylic acid, thalidomide, pranlukast, triflusal, glycyrrhizic acid, fish oil |
| SRC | dasatinib, citric acid, bosutinib, ponatinib, nintedanib, fostamatinib | | | | | | |
| IKBKB | mesalazine, sulfasalazine, acetylsalicylic acid, auranofin, arsenic trioxide, acetylcysteine, fostamatinib, | | | | | | |
| PRKCA | phosphatidyl serine, vitamin e, tamoxifen, ingenol mebutate, midostaurin, alpha-tocopherol succinate, d-alpha-tocopherol acetate, | | | | | | |
| IFNAR1 | peginterferon alfa-2a, interferon alfa-n1, interferon alfa-n3, peginterferon alfa-2b, interferon alfa-2a, recombinant, interferon beta-1a, interferon beta-1b, interferon alfacon-1, interferon alfa-2b, recombinant, natural alpha interferon | | | | | | |
| KDR | sorafenib, sunitinib, ramucirumab, pazopanib, midostaurin, axitinib, cabozantinib, regorafenib, ponatinib, lenvatinib, nintedanib, fostamatinib | | | | | | |
| VEGFA | bevacizumab, minocycline, gliclazide, carvedilol, ranibizumab, pidolic acid, tromethamine, vandetanib, dalteparin, aflibercept, chondroitin sulfate, foreskin keratinocyte (neonatal) | | | | | | |
| EGFR | cetuximab, trastuzumab, lidocaine, gefitinib, erlotinib, lapatinib, panitumumab, vandetanib, afatinib, osimertinib, necitumumab, foreskin keratinocyte (neonatal), icotinib, neratinib, fostamatinib, brigatinib, olmutinib, | | | | | | |
| TNF | etanercept, adalimumab, infliximab, chloroquine, epinephrine, pseudoephedrine, thalidomide, glucosamine, clenbuterol, pranlukast, amrinone, isopropyl alcohol, apremilast, golimumab, certolizumab pegol, pomalidomide, polaprezinc, foreskin fibroblast (neonatal), foreskin keratinocyte (neonatal), binimetinib, glycyrrhizic acid, | | | | | | |
| PTGS2 | dihomo-gamma-linolenic acid, icosapent, adapalene, aminosalicylic acid, mesalazine, acetaminophen, indomethacin, nabumetone, ketorolac, tenoxicam, lenalidomide, celecoxib, tolmetin, rofecoxib, piroxicam, fenoprofen, valdecoxib, diclofenac, sulindac, flurbiprofen, etodolac, mefenamic acid, naproxen, sulfasalazine, phenylbutazone, meloxicam, carprofen, diflunisal, suprofen, salicylic acid, meclofenamic acid, acetylsalicylic acid, bromfenac, oxaprozin, ketoprofen, balsalazide, thalidomide, ibuprofen, lumiracoxib, magnesium salicylate, salsalate, choline magnesium trisalicylate, ginseng, antrafenine, antipyrine, tiaprofenic acid, etoricoxib, resveratrol, niflumic acid, nimesulide, lornoxicam, aceclofenac, nepafenac, parecoxib, pomalidomide, loxoprofen, dexibuprofen, dexketoprofen, tolfenamic acid, morniflumate, propacetamol, talniflumate, phenyl salicylate, trolamine salicylate, menthyl salicylate, glycol salicylate, dipyrithione, alclofenac, bufexamac, acemetacin, fish oil, | | | | | | |
